# Cohabiting and becoming a parent: associations with changes in physical activity in the 1970 British cohort study

**DOI:** 10.1186/s12889-020-09187-2

**Published:** 2020-07-10

**Authors:** André O. Werneck, Eleanor M. Winpenny, Esther M. F. van Sluijs, Kirsten Corder

**Affiliations:** 1 MRC Epidemiology Unit and Centre for Diet and Activity Research (CEDAR), University of Cambridge School of Clinical Medicine, Institute of Metabolic Science, Cambridge Biomedical Campus, Box 285, Cambridge, CB2 0QQ UK; 2 Department of Physical Education, Universidade Estadual Paulista “Júlio de Mesquita Filho” (UNESP), Presidente Prudente, Brazil

**Keywords:** Exercise, Family, Parent, Adult, Child, Mother, Father

## Abstract

**Background:**

We examined the association between family–related life events (cohabitation/marriage and becoming a parent) and change in physical activity.

**Methods:**

Longitudinal data (*n* = 8045) from the 1970 British Cohort Study (30 and 34 years) were included. Life events (beginning cohabitation/marriage and becoming a parent) were reported and coded: 0 no, 1 yes, for each event occurring between 30 and 34 years. Participants reported frequency of participation in leisure-time physical activity at 30 and 34 years (Likert scale: mean change calculated ranging between − 4 and 4). Linear regression models were used to examine the association between life events and physical activity change (comparing individuals experiencing events between 30 and 34 years versus never experiencing the event - excluding participants that experienced previous events – with a final analysis sample of *n* = 3833 in parenthood analysis; *n* = 1137 in cohabitation analysis). Interaction terms were used to analyse combined parenthood and cohabitation status. Analyses were adjusted for level of education achieved, ethnicity, country of origin and other life events. ANCOVA was used to examine associations between change in physical activity and child age.

**Results:**

Compared to remaining without children, becoming a parent was associated with a greater reduction in physical activity among men [β:-0.234(95%CI:-0.396 to − 0.072)] but not women [0.126(− 0.048;0.301)]. No associations were found between cohabitation and physical activity. Men who became fathers both while cohabitating [− 0.201(− 0.383;-0.020)] and without cohabiting [− 0.937(− 1.623;-0.250)] experienced greater physical activity declines than those remaining single and without children; the decline was greatest among non-cohabiting fathers. These associations did not differ by child age.

**Conclusions:**

Parenthood appears to differentially impact physical activity for men and women; this association also differs by cohabitation status. Parenthood appears to be most detrimental to physical activity levels among men. Interventions for physical activity could target new or soon-to-be parents, especially fathers. Further analyses with device-measured physical activity data would be valuable to advance understanding of these associations.

## Background

Physical activity participation is protective of a wide range of health outcomes throughout the life course, including cardiovascular diseases, type 2 diabetes, cancer [1], mental illness [2, 3] and all-cause mortality [1]. Physical activity is also associated with a reduction in disability-adjusted life years worldwide [4]. Global estimates indicate that physical activity levels are low, with 27.5% of the adult population estimated to be inactive [5].

Participation in physical activity is reported to decline throughout adulthood, in response to biological, environmental and social influences [6, 7]. Particular life events may be associated with changes in the existing pattern of behaviours due to shifting demands and responsibilities [8]. The literature suggests the importance of cohabitation (including marriage) to health outcomes and longevity [9, 10]. It has been suggested that this may be because ‘healthier’ people are more likely to get married [11], or that cohabiting people may look after each other [12]. Moreover, these changes may differ by sex [13, 14]. Men may reduce risky behaviours (such as smoking) on getting married [15] but this may not apply to physical activity. The majority of studies have reported cohabitation to be negatively associated with long term activity levels in both sexes [13, 16], but findings are mixed: some present null associations [13, 17, 18] or even trends for positive associations [19], possibly dependent on age and life stage. The prospective association between cohabitation and physical activity is unclear and is likely to be complex due to several influential factors such as sex differences and differences between cultures [17, 20], and warrants further investigation. Moreover, the majority of previous studies have not focused on the comparison of data from before and after life events, taking into account the underlying trend in the population. As cohabitation often occurs irrespective of marriage, and most couples who marry cohabit prior to this [21], we refer to cohabitation and marriage together.

In addition, for both men and women, the onset of parenthood, while often joyful, is frequently accompanied by heightened stress and potentially harmful changes in behaviour including decreases in physical activity [22–25]. Such changes in behaviour over the transition to parenthood may have a long-lasting impact in shaping health trajectories into midlife [26].

Life events may be interrelated and are likely to have interactive effects on behaviour, for example the effect of parenthood on behaviour may depend on cohabitation status [27, 28]. Having a partner present in the home may allow childcare responsibilities to be shared, affording an individual more time for physical activity than if parenting alone [29]. This may also differ by sex, with single mothers shown to have lower mental health and higher social deprivation than single fathers [30], possibly associated with lower physical activity [3, 31]. Current research on the impact of cohabitation and parenthood on change in physical activity is inconclusive [13, 17, 18], and few studies have examined the impact of these life events in combination; the evidence assessing this is equivocal [13].

A better understanding of the impact of cohabitation and parenthood on physical activity could identify windows of opportunity for behaviour change, find groups at high-risk of large physical activity declines and consequently guide possible interventions. Therefore, our aim in this study was to examine the association between first starting cohabitation and becoming a first-time parent on changes in physical activity between 30 years and 34 years, and to examine whether cohabitation status impacts the association between becoming a first-time parent and change in physical activity and to investigate potential sex differences. Our hypothesis is that these life events are associated with larger declines in physical activity than remaining single and without children.

## Methods

### Cohort design and sample

The 1970 British Birth Cohort (BCS70) is a multidisciplinary longitudinal study. Initially, BCS70 was designed as the British Birth Survey and included all individuals from England, Scotland, Wales and Northern Ireland who were born in a specific week of 1970. These individuals have now been followed-up up to 9 times [32]. The present study analysed data from the 2000 and 2004 waves, which included questions regarding physical activity during adulthood. All questionnaire data was collected through face-to-face interviews. Participants provided consent during the interviews. Before the interviews, study members were sent an advance letter advising them about the survey. The letter was accompanied by detailed information about the survey and the cohort members were free to request further information, or to opt out of the survey at this point. Also, the cohort members could request further information or refuse involvement during all the survey process, including when the interviewer attempted to make an appointment to visit, when the interviewer visited and at any point during the administration of any elements of the surveys. The verbal consent was approved as it was a routine since the beginning of the cohort, in 1970 [33]. All procedures were approved by the London Multi-Centre Research at the 2000 wave (process 98/2/120) and by an internal committee from the Centre for Longitudinal Studies, Institute of Education, University of London, for the 2004 wave [33]. The majority of cohort members answered the questions in the second trimester of the year 2000 at baseline (41.2%), while the majority of cohort members were investigated at the first trimester of 2004 at follow-up (47.2%). The initial study sample was composed of 17,284 people (in 1970). Our final sample consisted of 8045 who completed the questionnaires at both ages 30 years and 34 years. As we aimed to investigate the impact of parenthood and cohabitation on physical activity, participants who became parents before 30 years (*n* = 4212) and/or who first started cohabitation before 30 years (*n* = 6908) were excluded from parenthood and cohabitation analyses respectively, therefore our analysis samples were 3833 participants for parenthood analyses and 1137 cohabitation analyses. A sensitivity analysis of change in physical activity and child’s age included 1054 participants (individuals who became a parent between 30 and 34 years).

### *Changes in* physical activity

Physical activity was assessed with a questionnaire designed for the study at 30 years and 34 years. Participants were first shown a list of leisure-time physical activity (1. Take part in competitive sport of any kind; 2. Go to “keep fit” or aerobics classes; 3. Go running or jogging; 4. Go swimming; 5. Go cycling; 6. Go for walks; 7. Take part in water sports; 8. Take part in outdoor sports; 9. Go dancing; 10. Take part in any other sport or leisure activity which involves physical exercise), and then asked: “Do you regularly take part in any of the activities? By regularly I mean at least once a month, for most of the year.” Possible answers were: “yes” or “no”. Participants who answered “yes” were also asked the Likert scale question: “How often do you take part in any activity of this type?” Possible answers were: “everyday”, “4 to 5 days a week”, “2 to 3 days a week”, “once a week”, “2 to 3 times a month”, or “less often”. Given that “2 to 3 times a month” and “less often” is less than once a week, we combined these categories with those who reported no exercise, creating five categories: “everyday”, “4 to 5 days a week”, “2 to 3 days a week”, “once a week” or “less often”. To estimate change in physical activity between 30 and 34 years-old, we calculated the difference between the two time points (physical activity at 34 minus physical activity at 30). Change scores ranged between − 4 and 4 to represent a 9-point Likert scale.

### Life events

To assess life events, we used questionnaires, in which participants reported if they were cohabitating or married and if they had a child. Cohabitation was first assessed at age 21 years, when the cohort member was asked: “Have you ever lived with anyone as a couple?” and “When did you start living with this person?”. Following this, a question was asked at each subsequent wave (26, 30 and 34 years) which assessed if the cohort member was married, cohabiting, single, separated, divorced or widowed (“Are you currently in a relationship with someone, whether or not you are living together?”). For parenthood, cohort members were asked: “Has anyone you were having a sexual relationship with ever become pregnant? / Have you been pregnant?” at 30 and 34 years. If the answer was “yes”, participants were also asked the year of birth of the child. From these data we created two dichotomous indicators: a) becoming a first-time parent between 30 and 34 years and b) first starting cohabitation between 30 and 34 years; coded as 1 for having experienced the life event and 0 if the individual had not experienced the event. Cohabitation status at 34 years (yes or no) was used in interaction analyses to examine whether the association of change in physical activity with becoming a first-time parent differed according to cohabitation status. For individuals becoming first-time parents between 30 and 34 years, age of child (when parent was 34 years, child age range 0–4) was estimated from parental report of child’s year of birth, reported at age 34. We tested the effect of reporting the birth of twins or more than one pregnancy between 30 and 34 years (also among participants without children before 30) on physical activity in sensitivity analyses.

### Covariates

Country of origin (England, Wales or Scotland) was derived from initial assessments of the birth cohort. Ethnicity was self-reported and classified as “British” or “Non-British”. Month of data collection at baseline and follow-up was included to adjust for seasonality. We assessed participant level of education achieved, using their highest qualification and categorized participants into the following four groups: None (no formal education or incomplete secondary education), low (general certificate of secondary education or certificate of secondary education), middle (at least one A level or diploma of higher education) and high (degree or higher degree). End of cohabitation was self-reported. Previous life events, including becoming a first-time parent and beginning cohabitation (before 30 years) were also included as covariates where relevant, for example, for the analysis examining change in physical activity when becoming a first-time parent, cohabitation status was included as a covariate, although those who had become a parent previously were excluded from that analysis.

### Statistical analysis

We used frequencies, means and standard deviations to describe the sample. Mann-Whitney tests and Chi-square tests were used to compare sample characteristics between men and women. All models linear regression models were conducted separately for men and women, due to differences by sex in physical activity levels and in the expected effect of life events on physical activity [13, 34]. Analyses were adjusted for level of education achieved, ethnicity, country of origin, the other life events where relevant (e.g. starting cohabitation for the association between becoming a first-time parent and change in physical activity), end of cohabitation between age 30 and 34, and previous life events when appropriate (e.g. starting cohabiting before 30 years in analyses of becoming a first-time parent). We examined the association between changes in physical activity (between 30 and 34 years) and a) starting cohabitation, and b) becoming a first-time parent (with the reference group as never having experienced that life event). Subsequently, interaction terms were included in the models of becoming a first-time parent to examine whether the associations of change in physical activity with new parenthood varied according to cohabitation status at 34 years. As a sensitivity analysis we examined the association between child’s age and change in physical activity for individuals who became a parent between 30 and 34 years, using estimated marginal means and 95% confidence intervals from analysis of covariance (ANCOVA). All analyses were conducted using the software Stata 15.1.

## Results

Characteristics of the sample are presented in Table 1. We report both the mean frequency of participation in leisure time physical activity and the percentage of the population reporting participation in leisure time physical activity four or more days/week at each wave, as well as the mean change in frequency of leisure time physical activity (as a change in likert scale score) between the two time waves. Women reported higher frequency of leisure time physical activity at both time points and also a slightly higher mean change in physical activity score between 30 and 34 years when compared with men (change in physical activity score: Women: 0.10 ± 1.81 vs. Men: − 0.03 ± 1.61; *p* < .001). Prevalence of starting cohabitation and becoming a first-time parent was similar for men and women, men had a higher prevalence of starting cohabitation than women (6.4% vs. 3.4%). For participants who became a parent, the mean age of their child at 34 years was 2.27 ± 0.95 among men and 2.35 ± 0.97 among women. Baseline values of physical activity frequency were not different between those that become parents or not, as well as between those who started cohabitation and who did not start cohabitation. In the restricted sample for parenthood analysis as well as cohabitation analysis, there was a higher proportion of men and participants with higher educational status, however, there were no substantial differences in ethnicity, country of origin and physical activity maintenance compared to the full sample.

**Table 1:** Characteristics of sample according to gender

	Full sample^a^			Parenthood analysis			Cohabitation analysis		
	Male (***n*** = 3766)	Female (***n*** = 4279)	***p***	Male (***n*** = 2139)	Female (***n*** = 1694)	***p***	Male (***n*** = 686)	Female (***n*** = 451)	***p***
*Country of birth*			.542			.769			.409
England	84.6%	84.6%		84.9%	85.4%		85.3%	82.5%	
Wales	5.6%	6.0%		5.4%	4.9%		5.7%	6.2%	
Scotland	9.8%	9.4%		9.7%	9.8%		9.0%	11.3%	
*Ethnicity*			.088			.391			.561
British	95.3%	96.1%		95.3%	95.9%		95.0%	95.8%	
Non-British	4.7%	3.9%		4.7%	4.1%		5.0%	4.2%	
*Level of education achieved*			<.001			<.001			.049
None	26.8%	23.8%		21.9%	15.9%		24.3%	25.9%	
Low	38.7%	40.2%		35.4%	33.0%		37.0%	29.3%	
Medium	11.9%	15.2%		12.9%	16.8%		11.1%	13.8%	
High	22.6%	20.8%		29.8%	34.3%		27.6%	31.0%	
**Physical activity**									
*30 years*									
Mean PA (Likert scale)	2.63 ± 1.37	2.72 ± 1.45	.037	2.72 ± 1.36	2.67 ± 1.33	.272	2.76 ± 1.41	2.62 ± 1.34	.123
Physical activity (at least 4 days/week)	25.4%	28.9%	< 0.001	27.0%	25.3%	.224	29.2%	24.4%	.078
*34 years*									
Mean PA (Likert scale)	2.60 ± 1.38	2.81 ± 1.45	<.001	2.67 ± 1.38	2.77 ± 1.39	.026	2.69 ± 1.41	2.68 ± 1.41	.893
Physical exercise (at least 4 days/week)	25.0%	31.2%	<.001	26.0%	29.3%	.025	27.6%	28.2%	.824
PA change between 30 and 34 years (Likert scale)	−0.03 ± 1.61	0.10 ± 1.81	.001	− 0.05 ± 1.57	0.10 ± 1.63	.001	−0.07 ± 1.63	0.06 ± 1.68	.175
**Life transition events**									
Becoming a parent between 30 years and 34 years	13.9%	12.4%	.050	24.5%	31.4%	<.001	5.7%	5.5%	.919
Start cohabiting between 30 years and 34 years	6.4%	3.4%	<.001	10.4%	6.9%	<.001	35.1%	32.4%	.337

Note. Values are presented using frequencies (percentages of sample), means and standard deviation. PA: physical activity. Parenthood analysis only includes those who become a first-time parent between 30 and 34 years vs. those who never had a child – those who become a parent before 30y were not included in this analysis. Cohabitation analysis only includes those who first started cohabitation between 30 and 34 years vs. never cohabiting – those who first started cohabitation before 30y were not included in this analysis. Level of education achieved categories: None: No formal education or less than secondary school. Low: Secondary school. Medium: At least one A level or diploma of higher education. High: Degree or higher degree. ^a^ Full sample includes all those with data available at age 30y and 34y

Table 2 presents the association between starting cohabitation or becoming a first-time parent and changes in physical activity frequency between 30 and 34 years. There was no association between starting cohabitation and change in frequency of physical activity in either men or women. However, becoming a first-time parent was associated with a slight [− 0.234 (95%CI: − 0.396 to − 0.072)] decrease in physical activity frequency score among men whereas no association was observed among women (0.126 (95%CI: − 0.048 to 0.301). In this sense, stratified analysis revealed that men that became a parent showed a reduction in the frequency of physical activity, while there was no change in physical activity frequency over time among non-parent men (**Supplementary Figure A**). Among women, both groups (becoming a first-time parent and non-parent) presented similar trends regarding change in physical activity frequency. Cohabitation was also not associated with change in physical activity frequency in either sex. Among parents, we found that having more than one child between ages 30 and 34 years [Men (*n* = 301): 0.109 (95%CI:-0.167 to 0.384); Women (*n* = 489): -0.046 (95%CI:-0.346 to 0.253)] was not associated with change in physical activity frequency. The proportion of twins was low (men: 25, female: 42) and twin parents were included as having more than one child.

**Table 2:** Association of parenthood and cohabitation with changes in physical activity (Likert scale ranging between − 4 and 4) between 30 and 34 years (*n* = 3833 for becoming a parent and *n* = 1137 for starting cohabitation)

	Male	Female
	β (95% CI)	β (95% CI)
Becoming a parent^a^	−0.234 (−0.396 to − 0.072)	0.126 (− 0.048 to 0.301)
Starting cohabitation^b^	0.043 (− 0.240 to 0.326)	−0.012 (− 0.375 to 0.349)

Note. ^a^Only included those who become a first-time parent between 30 and 34 years vs. non-parents (Male: *n* = 2139; Female: *n* = 1694). ^b^Only included those who first started cohabitation between 30 and 34 years vs. never cohabiting (Male: *n* = 686; Female: *n* = 451). Models were adjusted for level of education achieved, ethnicity, country of origin, end of cohabitation, month of data collection at baseline and follow-up, other life events (e.g. start cohabiting for the association between becoming a parent and physical activity) and previous life events (first starting cohabitation and have a child before 30y). *CI* Confidence interval. Reference group is individuals who have never experienced that transition

When examining whether the association between change in physical activity frequency and parenthood differed by cohabitation status, evidence of an interaction was present for men but not women (Table 3). Men who became fathers while cohabitating [β:-0.201 (95%CI:-0.383 to − 0.020)] and without cohabiting [β:-0.937 (95%CI:-1.623 to − 0.250)] both experienced slightly greater declines in physical activity than non-cohabiting non-fathers, but the decline for non-cohabiting fathers appeared larger.

**Table 3:** Combined associations of parenthood and cohabitation with changes in physical activity (Likert scale ranging between − 4 and 4) between 30 and 34 years (*n* = 3833)

	Male	Female
	β (95% CI)	β (95% CI)
No child / no cohabitation	Ref	Ref
No child / starting cohabitation	−0.033 (− 0.188 to 0.123)	0.020 (− 0.175 to 0.215)
Becoming a parent / no cohabitation	−0.937 (−1.623 to − 0.250)	−0.264 (− 0.940 to 0.411)
Becoming a parent / starting cohabitation	− 0.201 (− 0.383 to − 0.020)	0.154 (− 0.056 to 0.364)

Note. Only included those who become a parent between 30 and 34 years vs. non-parents. Multiplicative interaction for male: 0.768 (0.061 to 1.476); *p* = 0.033. Multiplicative interaction for female: 0.398 (− 0.302 to 1.099); *p* = 0.265. Models adjusted for level of education achieved, ethnicity, country of origin and month of data collection at baseline and follow-up. *CI* Confidence interval

**Fig. 1:**
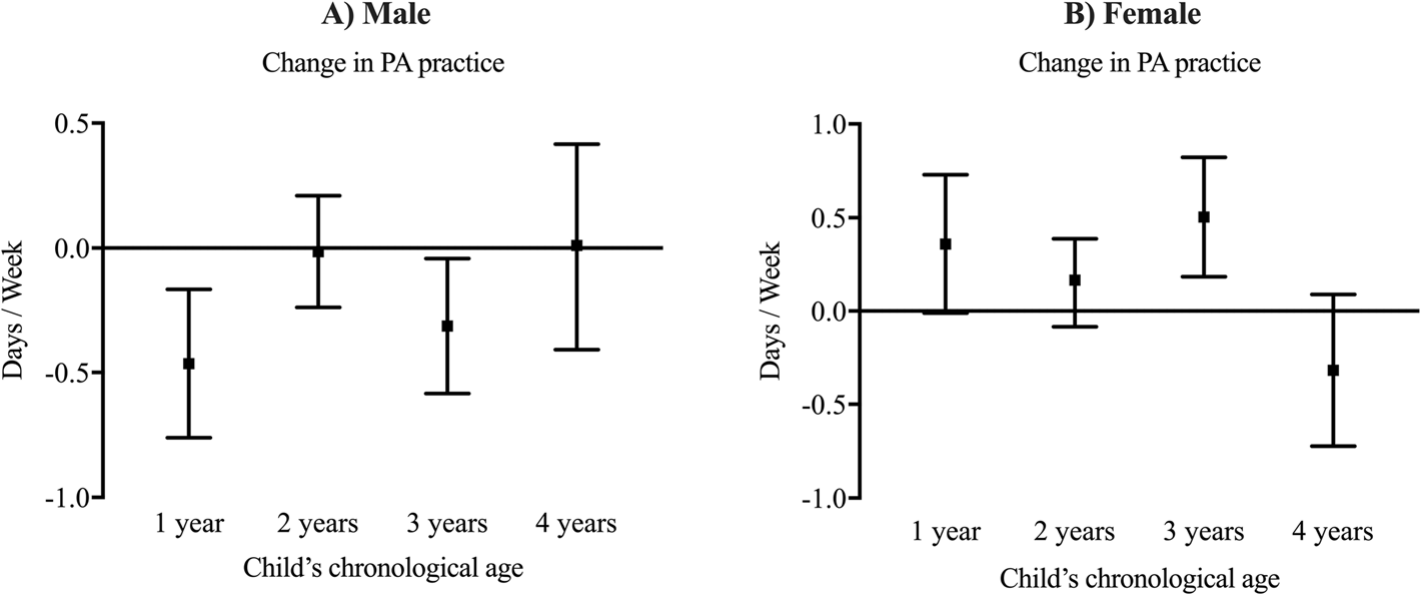
Mean changes in physical activity (Likert scale ranging between − 4 and 4) according to child’s chronological age between 30 and 34 years (Men: *N* = 520; Female: *N* = 531). Note. Values are presented using estimated marginal means and 95% confidence intervals. Models were adjusted for level of education achieved, ethnicity, country of origin, month of data collection at baseline and follow-up and starting cohabitation. PA: physical activity. Values: Male = 1 year: -0.464 (95%CI: − 0.762 to − 0.166); 2 years: -0.014 (95%CI: − 0.238 to 0.210); 3 years: -0.313 (95%CI: − 0.585 to − 0.042); 4 years 0.011 (95%CI: − 0.408 to 0.415). Female = 1 year: 0.360 (95%CI: − 0.011 to 0.730); 2 years: 0.165 (95%CI: − 0.085 to 0.387); 3 years 0.504 (95%CI: 0.184 to 0.824); 4 years − 0.318 (95%CI: − 0.724 to 0.089).

For individuals who became parents between 30 and 34 years, sensitivity analysis of whether changes in frequency of physical activity differed according to child’s age revealed no consistent associations among men or women; patterns of association are displayed graphically in Fig. 1.

## Discussion

### Main findings

The impact of parenthood on change in physical activity frequency appears to differ for men and women and according to cohabitation status. Among men, becoming a first-time father between ages 30 and 34 years was associated with a slightly greater decline in physical activity compared to men without children; no difference was seen among women. Non-cohabiting fathers experienced a slightly greater decline in physical activity frequency compared to cohabiting fathers and non-parents. Beginning cohabitation (without parenthood) was not associated with change in physical activity for men or women, when compared to non-cohabiting individuals. The association between becoming a first-time parent and change in physical activity did not differ by child age for either mothers or fathers.

### Comparison with previous evidence

We did not find an association between starting cohabitation and change in physical activity, which aligns with previous evidence which shows inconsistent associations [17, 18]. It has been suggested that people are likely to marry those with similar characteristics, which may extend to physical activity, and it is plausible that this is also relevant to cohabitation [35]. If two cohabiting individuals have similar physical activity levels prior to living together, it may be relatively unlikely that they change behaviour when cohabiting. Increased social responsibilities related to cohabitation may reduce time available for physical activity, but conversely, the presence of an active partner could positively influence activity levels [36]. This could mean that conflicting directions of associations could cancel each other out statistically, masking the myriad of contradictory effects related to complex social changes entailed by beginning cohabitation.

Becoming a first-time father was associated with greater physical activity declines than among men without children, which is somewhat consistent with previous literature, which mainly indicates a reduction in physical activity with parenthood [13, 28, 37]. Our findings agree with a previous study which found that the impact of parenthood was higher among fathers, especially for those having their first child [17]. One explanation suggested was that this might be due a higher physical activity among men before fatherhood and a consequently higher decline in comparison with women [17], however, our own results showed a similar frequency of physical activity by sex at baseline. Therefore, further investigation of the potential mediators of the effect of becoming a parent on physical activity is warranted.

Traditionally in the UK, women take on more child care responsibility than men [38], which we hypothesize may make it particularly difficult for mothers to participate in leisure-time physical activity. Our results indicated that among women becoming first-time mothers, no difference in change in physical activity was seen compared to women who remained without children. Incidental activities involved with childcare such as walking with a buggy or pram, ‘chasing around’ after a child or co-participation in activity with children, may increase in frequency as a parent even though they may be conducted at a relatively low intensity [39, 40]. Time taken away from work for maternity leave or childcare may be higher among women than men and may increase opportunities for these types of physical activity.

Our results indicate the complexity of the combined effect of becoming a first-time parent and cohabitation on physical activity [13]. It has been suggested that becoming a first-time parent may interact with, or may mediate, the association between starting cohabitation and physical activity [13]. This aligns with our finding of greater physical activity declines among non-cohabiting men, with cohabiting men experiencing a smaller decline in physical activity when becoming a first-time father. However, the direction of the association that we identified was unexpected as we assumed that cohabiting men are more likely to live with their child and that therefore, there may be a greater time commitment of parenthood for co-habiting versus non-cohabiting fathers. Nevertheless, we were not able to establish whether the child lives full-time with their father in order to further explore this. Further investigation of the impact of fatherhood and cohabitation on physical activity, including measures of child living arrangements and incorporating qualitative methods to explore context, would help gain a deeper understanding of these findings.

### Strengths and limitations

We examined change in the frequency of physical activity over the transition of becoming a first-time parent, whereas much previous evidence has compared physical activity levels between parents and non-parents without examining activity levels before parenthood [28, 41]. This methodological difference may at least partly explain differences between our results and cross-sectional reviews which often indicate that physical activity levels are lower for both mothers and fathers than individuals without children [28, 41]. Further examination of the effect of parenthood on a spectrum of physical activity intensities and related behaviours, including the use of device-measured physical activity assessment, would be valuable to further understand the impact of parenthood on behaviour and health.

Our findings should be interpreted in the light of limitations. Firstly, we used a small sub-sample of participants due to our research question, these may not be representative of the original participants and consequently may bias the findings. We aimed to examine change in activity levels over the transition to parenthood and the start of cohabitation, therefore it was appropriate to use data which allowed a comparison of the impact of these events compared to individuals who had not experienced them, even though this resulted in a reduced sample size. Our sample was restricted to those who began cohabiting or became a parent after age 30. The average age of parenthood in the UK was 28.5 years and 31.7 years for mothers and fathers respectively in the year 2000 (when these data were collected) [42] and the average age at the first marriage was 29.6 years for men and 27.5 for women [43], so our sample are slightly older than the average age for these life events. We presented self-reported measures of physical activity in one domain (leisure), using a single question regarding frequency of physical activity, without consideration of intensity of physical activity; all physical activity questionnaires [44, 45] are prone to reporting bias, however, device-measured physical activity data were not available. This question has been used previously [7, 46–49], but has its validity and reliability are unknown. We derived a nine-point change score using the Likert scales from the physical activity questions, which do not necessarily represent exact frequency in days/week of activity. However, physical activity questionnaires may be more suitable to rank individuals’ physical activity levels than to quantify frequency or duration of activity [50, 51]. The same assessment method of physical activity was used at baseline and follow-up, and assuming that any bias would be the same at both measurement waves, this bias would not affect the change in response between waves, the outcome in this analysis. There may be unmeasured confounding or potential mediators that we were not able to consider in the analyses such as sedentary behaviour, family support and income. Finally, we acknowledge that the data was collected some time ago, but this is unlikely to have affected the associations of interest. To our knowledge there are no recent national cohorts with device-measured physical activity data considering the specific age group of the present study and collecting detailed data on life events.

Despite these limitations, these analyses made use of prospective data from a large national British birth cohort with data on the association between change in physical activity and family-related life events, which is the most appropriate data available to analyse our research questions. There has been little analysis of longitudinal cohort data to address our research questions [14] and to our knowledge, these have not previously been examined in a British population. Despite the limitations of self-reported physical activity data, this is more feasible to collect longitudinally than device-measured physical activity data, especially considering the wide range of variables collected by the BCS70 over the study years.

## Conclusion

Beginning first cohabitation was not associated with changes in physical activity, while the impact of parenthood on change in physical activity appears to differ for men and women and according to cohabitation status. Having a child was associated with slightly greater declines in physical activity among men compared to those without children, particularly those men not living with a partner. Conversely, no declines in physical activity were identified for women associated with becoming first-time mothers. Future studies using device-measured assessment of physical activity and life events, including data across multiple time-points throughout adulthood in the UK, would be helpful to further investigate the detailed associations between life events such as parenthood and cohabitation on physical activity and related behaviours.

## Supplementary information

**Additional file 1: Supplementary Figure. A**. Mean changes in physical activity (Likert scale ranging between − 4 and 4) according to parenthood and cohabitation status. Note. Values are presented using estimated marginal means and 95% confidence intervals. Models were adjusted for level of education achieved, ethnicity, country of origin, month of data collection at baseline and follow-up and starting cohabitation for becoming a first-time parent analysis / becoming a first-time parent for starting cohabitation analysis. PA, physical activity. Values: Graph A: Male: non-parent: 0.007 (95%CI: − 0.077 to 0.090), becoming a parent: -0.191 (95%CI: − 0.340 to − 0.090). Female: non-parent: 0.061 (95%CI: − 0.042 to 0.163), becoming a parent: 0.211 (95%CI: 0.060 to 0.362). Graph B: Male: no cohabiting: -0.121 (95%CI: − 0.289 to 0.048), cohabiting: 0.008 (95%CI: − 0.219 to 0.234). Female: no cohabiting: 0.065 (95%CI: − 0.146 to 0.276), cohabiting: 0.058 (95%CI: − 0.244 to 0.360).

## Data Availability

Cohort data comply with ESRC data sharing policies, readers can access data via the UK Data Archive (www.data-archive.ac.uk), through a formal request.

## Abbreviations

UK: United Kingdom
ANCOVA: Analysis of covariance
BCS70: 1970 British birth cohort
CI: Confidence interval
