# Supplementary figures and images for: Cohabiting and becoming a parent: associations with changes in physical activity in the 1970 British cohort study
Source: BMC Public Health. 2020 Jul 10;20:1085. doi: 10.1186/s12889-020-09187-2 (PMC7353783; doi:10.1186/s12889-020-09187-2)

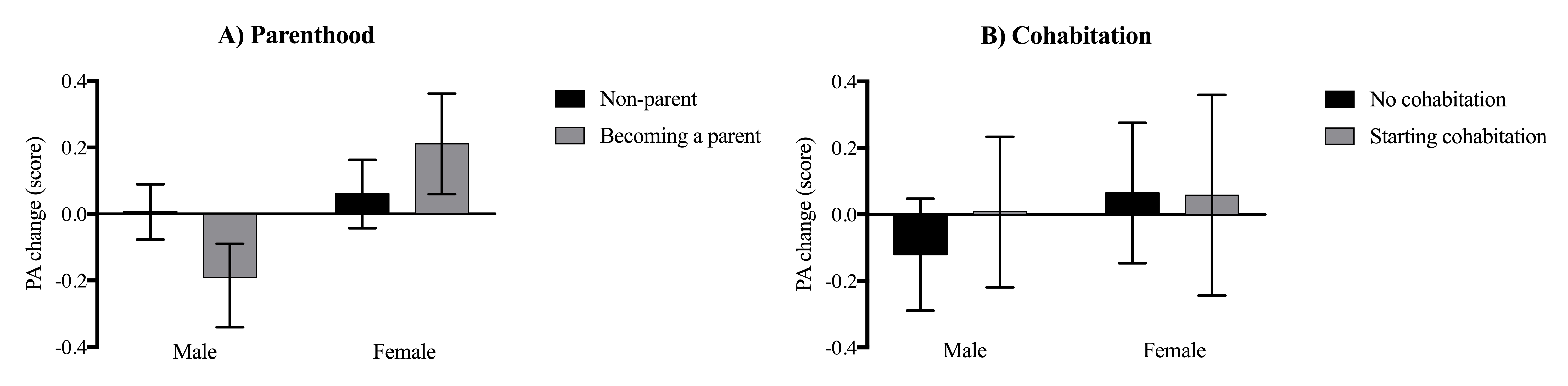

Supplement: Supplementary file 1 — Additional file 1: Supplementary Figure. A. Mean changes in physical activity (Likert scale ranging between − 4 and 4) according to parenthood and cohabitation status. Note. Values are presented using estimated marginal means and 95% confidence intervals. Models were adjusted for level of education achieved, ethnicity, country of origin, month of data collection at baseline and follow-up and starting cohabitation for becoming a first-time parent analysis / becoming a first-time parent for starting cohabitation analysis. PA, physical activity. Values: Graph A: Male: non-parent: 0.007 (95%CI: − 0.077 to 0.090), becoming a parent: -0.191 (95%CI: − 0.340 to − 0.090). Female: non-parent: 0.061 (95%CI: − 0.042 to 0.163), becoming a parent: 0.211 (95%CI: 0.060 to 0.362). Graph B: Male: no cohabiting: -0.121 (95%CI: − 0.289 to 0.048), cohabiting: 0.008 (95%CI: − 0.219 to 0.234). Female: no cohabiting: 0.065 (95%CI: − 0.146 to 0.276), cohabiting: 0.058 (95%CI: − 0.244 to 0.360). [file 12889_2020_9187_MOESM1_ESM.tiff]
